# Salivary psoriasin (S100A7) correlates with diffusion capacity of carbon monoxide in a large cohort of systemic sclerosis patients

**DOI:** 10.1186/s12967-016-1023-5

**Published:** 2016-09-08

**Authors:** Laura Giusti, Francesca Sernissi, Elena Donadio, Federica Ciregia, Camillo Giacomelli, Gino Giannaccini, Maria Rosa Mazzoni, Antonio Lucacchini, Laura Bazzichi

**Affiliations:** 1 Department of Pharmacy, University of Pisa, Via Bonanno 6, 56126 Pisa, Italy; 2 Department of Clinical and Experimental Medicine, University of Pisa, Via Roma 67, 56126 Pisa, Italy

**Keywords:** Systemic sclerosis (SSc), Psoriasin, S100A7, Whole saliva, Biomarker, Diffusion capacity of carbon monoxide (DLCO)

## Abstract

**Background:**

Systemic sclerosis (SSc) is an autoimmune disease characterized by progressive fibrosis of the skin and the internal organs. In a previous work we suggested a correlation between levels of salivary psoriasin (S100A7) and pulmonary involvement in SSc patients. The goals of this study are to determine the distribution characteristics of psoriasin in whole saliva (WS) of SSc and healthy donor populations and define its predictive value on diffusion capacity of carbon monoxide (DLCO), along with others clinical parameters.

**Methods:**

Salivary level of psoriasin was determined by ELISA kit in 134 SSc patients, 63 Raynaud syndrome patients, 40 patients affected by other connective diseases (non-case) and 74 healthy control subjects.

**Results:**

A significant increase of salivary psoriasin was observed in SSc patients when compared with other healthy and pathological controls. Moreover, we confirmed the efficacy of salivary psoriasin to correlate with DLCO in a large cohort of SSc patients.

**Conclusions:**

Overall our results suggest a rapid, non invasive and low costing method which can help clinicians in the evaluation of SSc pulmonary involvement.

## Background

Human psoriasin (S100A7) is a small calcium binding protein which belongs to the multigenic S100 family of EF-hand proteins [1, 2]. Human psoriasin was originally identified as an 11.4 kDa protein upregulated in inflamed hyperplastic psoriatic skin [3] where it acts as chemoattractant for T-cells to amplify inflammatory response in psoriasis. Recently, an highly homologous (93 % of identity) protein, koebnerisin (S100A15) [4], was also identified as up-regulated in psoriasis, thereby suggesting a role in the inflammatory phenotype. Nonetheless, a recent work of Wolf and coworkers [4] reveals an unexpected biological diversity into properties and functional mechanisms of psoriasin and koebnerisin to induce inflammation suggesting a functional synergism in inflammatory diseases. In fact, despite their high homology, both antimicrobial peptides psoriasin and koebnerisin are differentially regulated by different cell types in the skin and have distinct proinflammatory functions and mechanisms of action [5]. Nonetheless they acts synergistically as “alarmins” to prime skin cells for production of immunotropic cytokines, particularly the Th17 cytokines, that further amplify the inflammatory response. On the other hand, recent researches on Th17 function have associated cytokines in the development and pathogenesis of autoimmune diseases such as SSc and rheumatoid arthritis (RA) [5]. Focusing on pulmonary manifestations of SSc disease serum levels of some cytokines, i.e. IL-17, IL-21 and IL-23, [6] and serum amyloid A [7] have been linked to lung involvement and have been suggested as potential biomarkers for this complication.

In a previous study using a proteomic approach, we have shown an association of salivary psoriasin with SSc clinical manifestations suggesting 12 kDa psoriasin as a potential predictor of pulmonary involvement [8]. The novelty of our findings was the detection of marker in whole saliva (WS) whose sampling is a noninvasive, simple, safe and stress-free procedure that can be applied to large groups of subjects. Starting from preliminary results, in this work we extended the evaluation of the presence of salivary psoriasin and its correlation with clinical parameters in a large cohort of SSc patients. Therefore the aims of this study were: (1) to determine salivary psoriasin concentrations by a specific ELISA kit; (2) to define the distribution characteristics of salivary psoriasin levels in SSc patient and healthy subject populations by SPSS statistic analysis; (3) to confirm the correlation of the presence of salivary psoriasin with pulmonary involvement in the SSc population.

## Patients and methods

### Patients

One hundred thirty-four consecutive adult patients with diagnosis of SSc (16 male, 118 female; median age 55.4 ± 13.1 years), enrolled between January 2009 and June 2011, were included in the study. The study was approved by Local Ethics Committee. All patients met the ACR criteria [9]. WS samples were obtained at scheduled visits after patients signed consent form approved by Local Ethics Committee. WS was also collected from sixty-three patients with Raynaud syndrome (8 male, 55 female; median age 51.3 ± 16.7 years), forty patients affected by other connective diseases (non-case) (20 male, 20 female; median age 54.8 ± 12.1 years) and seventy-four healthy volunteers (14 male, 60 female; median age 40.2 ± 20.7 years). The clinical features of SSc patients are summarized in Table 1. Patients affected by Raynaud’s syndrome (24 primary and 30 secondary) and noncase patients (8 Undifferentiated Connective Tissue Disease (UCTD), 2 Sjogren’s syndrome, 17 psoriasis, 4 morphea, 4 polymyalgia, 1 myositis, 2 dermatomyositis, 1 rheumatoid arthritis, 1 microangiopathy), represented the pathological controls.

**Table 1:** SSc patient’s clinical and serological features

Features	Data (%)
No. of patients	134
Age, years (mean ± SD)	55.4 ± 13.1
Gender (M, F)	16 M, 118 F
Diffuse	44 (38)
Limited	71 (62)
Scl-70	36 (27)
ACA	47 (36)
FAN Hep2	109 (83)
Acral ulcers	28 (24)
Skin	74 (87)
Rodnan skin score	9 ± 7.3
Pattern capillaroscopy	87 (78)
Heart involvement	22 (19)
Kidney involvement	8 (7)
DLCO <70	49 (42)
Lung fibrosis (HRCT)	58 (47)
Pulmonary hypertension	11 (9)

*DLCO* diffusion capacity of carbone monoxide, *HRCT* high resolution computed tomography

### Ethics statements

The research was carried out according to The Code of Ethics of the World Medical Association (Declaration of Helsinki), and the author’s institutional review board has approved the study. This study was approved by the Local Ethics Committee (Comitato per la Sperimentazione Clinica dei Farmaci, Azienda Ospedaliera Universitaria Pisana) and signed consent forms were obtained from all patients before their enrolment in the study.

### Sample collection and preparation

WS samples were collected early in the morning in standard conditions, as previously described [10]. Briefly, in order to minimize protein degradation, the samples were processed immediately and kept on ice during the process. To remove debris and cells, centrifugation at 14,000*g* for 30 min at 4 °C was performed and samples were stored at −80 °C until assayed.

### Salivary psoriasin assay

The concentration of S100A7 in WS was detected by enzyme linked immunosorbent assay (ELISA) kit (*CircuLex*™, cat# CY-8073) according to manufacturer’s instructions. Briefly, samples (1:4000 dilution) and standards (ranging from 0.12 to 90 ng/ml) were added in the wells. After incubation and washing, horseradish peroxidase (HRP) conjugated antibody was added, incubated and then wells were washed before the addition by substrate reagent. The reaction was stopped and the plate read at 450 nm using a microplate-reader Wallac Victor 1420 (PerkinElmer, San Diego, CA, USA) The limit of detection was better than 0.12 ng/ml of sample.

### Statistical analysis

The normality of distribution of psoriasin levels was determined by Kolmogorov–Smirnov test. Due to non-normal distribution of the data, non parametric test (Mann–Whitney U test) was used to compare psoriasin levels. Spearman’s rank correlations were calculated to determine the correlation between psoriasin levels and various clinical and laboratory parameters. Data were analyzed using IBM SPSS Statistics Version 19. p < 0.05 was considered statistically significant. Receiver operating characteristic (ROC) curve was performed to assess the sensitivity and specificity (IBM SPSS Statistics Version 19).

## Results and discussion

The current report focuses on salivary levels of psoriasin protein in patients with SSc and their association with clinical and serological parameters, particularly with lung impairment. Pulmonary involvement is the most common manifestation of SSc. Usually, measurement of carbon monoxide diffusing capacity (DLCO), together with high-resolution computed tomography (HRTC), are initial pulmonary function tests in the evaluation of lung impairment. Nonetheless, recently Suliman and coworkers [11] have shown that the use of only pulmonary function tests as the screening method for SSc-interstitial lung disease, gives a high risk of missing significant rate of false–negative results. Previously, using a proteomic approach, we have shown several S100 proteins are altered in SSc WS including S100A7 (psoriasin) [8, 12]. In particular, we have shown that high levels of salivary psoriasin correlate with the severity of lung involvement of SSc patients. Likely, also Lakota et al. [7] have shown a correlation between high levels of serum amyloid A protein and pulmonary involvement in SSc suggesting a potential role of this protein as biomarker for this complication.

**Fig. 1:**
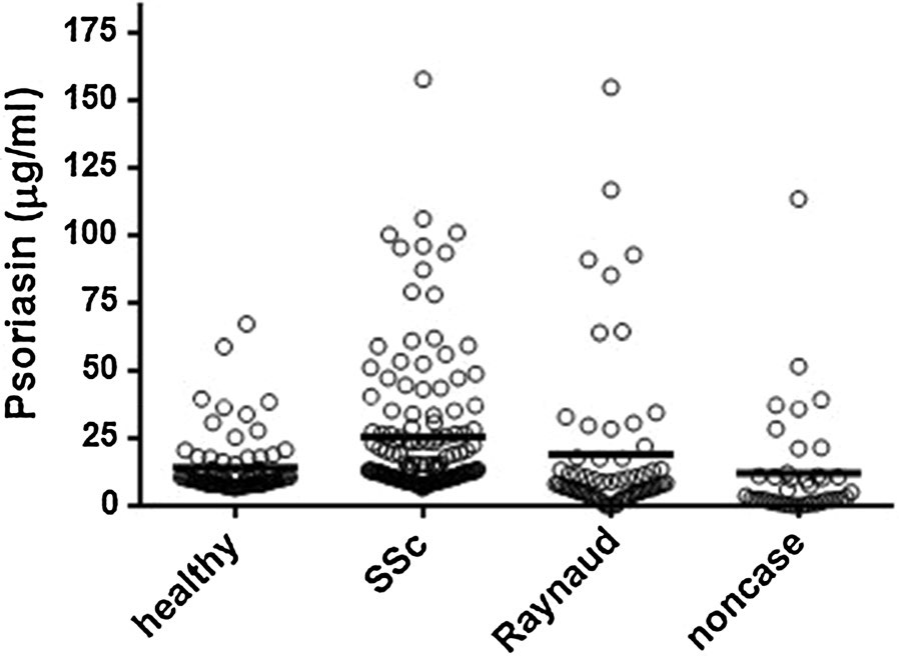
Psoriasin level were determined in SSc patients, healthy subjects and pathological controls. *Bold horizontal lines* represent the mean value

**Fig. 2:**
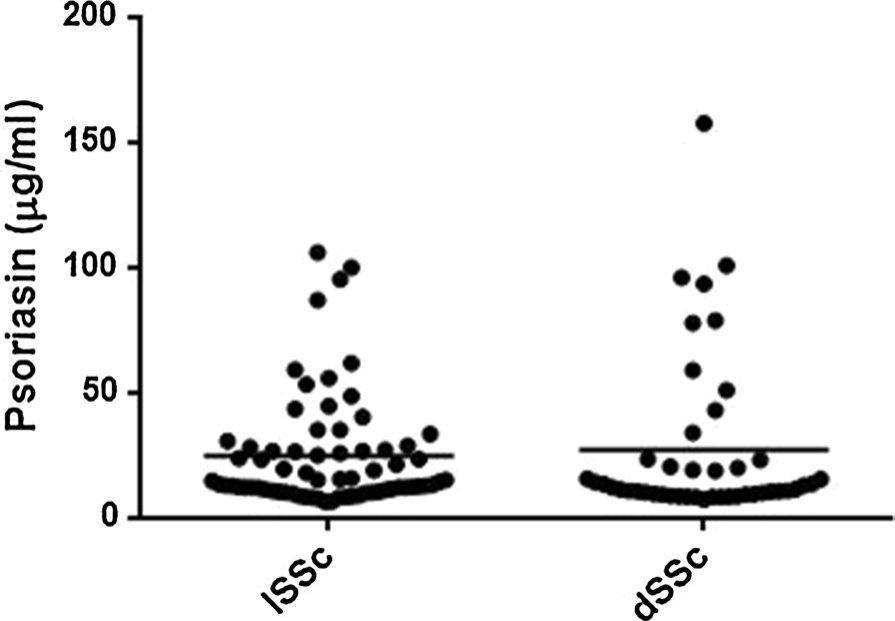
Psoriasin levels in the clinical SSc subtypes. *Bold horizontal lines* represent the mean value

**Fig. 3:**
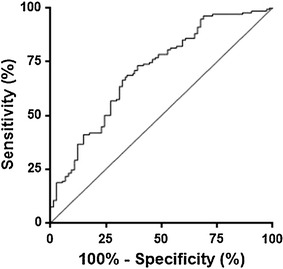
Receiver operate characteristic (ROC) curve of psoriasin in SSc vs healthy subjects

In this study, using an ELISA test, a quantification of psoriasin level in WS of SSc patients compared with that of healthy and pathological control subjects was performed. Psoriasin levels were analyzed using SPSS in different groups of subjects and a non normal distribution was observed. The mean values ± SE were 14.1 ± 1.3, 25.5 ± 2.2, 19.0 ± 3.8 and 12.1 ± 3.3 µg/ml for healthy, SSc, Raynaud and noncase groups, respectively. Figure 1 shows the scatter dot plot of psoriasin concentrations in different classes of patients. Highly significant difference (p < 0.0001****) of expression was observed in SSc with respect to all other groups. No significant difference in psoriasin levels were detected between two clinical subtypes [diffuse SSc (dSSc) and limited SSc (lSSc)] (p = 0.73) (Fig. 2). The diagnostic power of psoriasin has been also analyzed. ROC curve was calculated to evaluate the ability of psoriasin to separate healthy and SSc groups (Fig. 3). The sensitivity and specificity of psoriasin were 68 and 66 %, respectively and the derived area under curve (AUC) was 0.71. Moderate values of sensitivity and specificity derived from the ROC analysis performed in a large cohort of SSc patients, confirmed our previous observation that psoriasin is not suitable as early diagnostic biomarker of SSc [8]. However, Spearman’s analysis showed a direct correlation of psoriasin level with the presence of serological ACA (r = 0.264, p = 0.002**) and with DLCO (r = 0.186, p = 0.045*). No significant correlations were detected for psoriasin with other clinical and serological parameters taken in consideration (Table 2). Although we did not observe a statistically significant correlation of psoriasin levels with HRCT, however the significant correlation with DLCO, a functional pulmonary parameter, suggests that the assay of psoriasin in saliva might be a non invasive tool useful to strengthen the outcome of SSc. The pathogenetic relationship between psoriasin and lung impairment is still far from being completely clarified. As far as psoriasin is concerned different intracellular and extracellular functions have been suggested for this protein, including regulation of calcium homeostasis, cell proliferation, differentiation, apoptosis, cell invasion and motility, cytoskeleton dynamics, protein phosphorylation, regulation of transcriptional factors, immune responses, chemotaxis, antimicrobial, and inflammation [13]. In particular, a role of chemoattractant agents which are able to stimulate the neutrophyl and CD4^+^ T lymphocyte infiltration has been hypothesized by us to explain the link between psoriasin and lung involvement [8]. At this regard recently, the roles of Th17 and regulatory T (Treg) cells, in the autoimmune disease pathogenesis and in particular in SSc have been investigated [14]. In fact, Jiang and coworkers have shown that elevated Treg (CD4, CD25 and Foxp3 positive) cells are observed in SSc patients with a high interstitial lung disease score and low DLCO value. Moreover, the authors have suggested that Th17 cells participate in both inflammation and fibrosis by secreting IL-17, which is a potent inducer of several antimicrobial peptides and proteins such as psoriasin [14]. Therefore, we reinforce our previous suggestion that the increase of psoriasin level observed in SSc patients could be linked with T cell abnormalities and consequent alteration in the secretion of peculiar cytokines which are leading factors SSc pathogenesis and lung impairment [6, 14].

**Table 2:** Clinical correlations

Non-parametric correlations	Psoriasin (µg/ml)	Acral ulcers	Skin	ACA	SCL70	DLCO	Lung fibrosis (HRTC)
Rho Spearman							
Psoriasin (µg/ml)							
r	1.000	0.032	0.019	*0.264*	−0.150	*0.186*	0.117
p value	0.0	0.730	0.860	0.002**	0.084	0.045*	0.199
n	134	121	90	133	133	117	122
Acral ulcers							
r	0.032	1.000	0.219	0.053	0.093	0.186	0.132
p value	0.730	0.0	0.039*	0.566	0.311	0.053	0.161
n	121	121	89	121	121	109	114
Skin							
r	0.019	0.219	−	−0.128	0.089	0.256	0.095
p value	0.860	0.039*	0.0	0.228	0.406	0.019*	0.389
n	90	89	90	90	90	84	85
ACA							
r	0.264	0.053	−0.128	1.000	−0.467	−0.164	−0.336
p value	0.002**	0.566	0.228	0.0	0.000**	0.078	0.000**
n	133	121	90	133	133	117	122
SCL70							
r	−0.150	0.093	0.089	0.467	1.000	0.265	0.350
p value	0.084	0.311	0.406	0.000**	0.0	0.004**	0.000**
n	133	121	90	133	133	117	122
DLCO							
r	0.186	0.186	0.256	−0.164	0.265	1.000	0.451
p value	0.045*	0.053	0.019*	0.078	0.004**	0.0	0.000**
n	117	109	84	117	117	117	113
Lung fibrosis (HRTC)							
r	0.117	0.132	0.095	0.336	0.350	0.451	1.000
p value	0.199	0.161	0.389	0.000**	0.000**	0.000**	0.0
n	122	114	85	122	122	113	122

The significant correlations of psoriasin with clinical parameters are in italics

*r* correlation coefficient, *n* number of subjects, *DLCO* diffusion capacity of carbon monoxide, *HRCT* high resolution computed tomography

Statistical significance level: * p < 0.05

** p < 0.01

Overall our results confirm the increase of psoriasin in WS of SSc patients also when compared with pathological controls and its correlation with DLCO and ACA but not with the presence of anti-Scl-70 antibodies (p = 0.089) and lung fibrosis (p = 0.199) as previously suggested [8].

## Conclusions

The strength of our study includes the detection of marker in WS whose sampling is a noninvasive, simple, safe and stress-free procedure that can be applied to large group of subjects. On the other and the power of saliva as potential diagnostic fluid is largely documented considering numerous advantages with respect the serum [15–18]. Moreover, the assay of salivary psoriasin could be a rapid method allowing clinicians to assess the pulmonary involvement, which until now is revealed only by the use of more invasive and expensive instrumental investigations. Finally, we think that a future development could be the introduction of a point of care test for the realization of a rapid, simple, low-cost and accurate method of psoriasin directly from saliva.

## Abbreviations

SSc: systemic sclerosis
WS: whole saliva
DLCO: diffusion capacity of carbon monoxide
RA: rheumatoid arthritis
UCTD: undifferentiated connective tissue disease
ELISA: enzyme linked immunoassorbent assay
HRP: horseradish peroxidase
ROC: receiver operating characteristic
HRTC: high-resolution computed tomography
AUC: area under curve
dSSc: diffuse SSc
lSSc: limited SSc
